# Diagnosis and Treatment of Diseases of the Rectum, Anus, and Contiguous Textures

**Published:** 1897-06

**Authors:** 


**Diagnosis and Treatment of Diseases of the Rectum, Anus, and Contiguous Textures.** By S. G. Gant, M.D. Pp. xiv., 399. Philadelphia: The F. A. Davis Company. 1896.

Dr. Gant's volume is not undeserving of some amount of praise. It is printed on good paper, in clear bold type, and the numerous sub-divisions are made evident at a glance, by the device of separate paragraphs with headings, so that reference is greatly facilitated.

Fistula in ano is treated well, and the illustrations are useful from a diagrammatic point of view. Much stress is laid on the possibility of incontinence of fæces resulting even after a single division of the two sphincters, and on its extreme probability when the division is made in more than one place. The directions for the treatment of ramifying, multiple and horseshoe fistulæ are very practical; and the question of phthisis with regard to operation in these cases is considered. On this point Dr. Gant distinguishes between simple fistulæ in phthisical patients and tubercular fistulæ. Hemorrhoids are fully treated (with the exception of their pathology), and the author expresses his preference for the clamp and cautery, and gives fifteen reasons in favour of the superiority of his own clamp from its being " neat and attractive " to " the best clamp made, for the reason that it exerts equal pressure at all points and under all conditions.' On constipation the author has much to say, and much that is very true. He pins his faith to what he calls the non-medicinal method, which includes divulsion of the sphincter, abdominal massage, copious warm water injections, regularity in going to stool, dieting, free water-drinking, and other items of less universal applicability.

The numerous wood-engravings in the book are of average merit; but the large chromo-lithographs are hard and crude in outline and colouring, and the majority serve no useful purpose, while the few that might have been helpful are very atypical, and poor representations of the conditions supposed to be illustrated. The zeal for tabular classification has been carried to excess, and becomes wearisomely formal; moreover, it has led in some cases to the inclusion of trivial items, and in others to an imperfect elucidation of points of real importance. Of these defects, the chapter on general symptomatology is one of the most glaring examples, and the result is disappointing and practically unhelpful; it is over-burdened and inadequate. The congenital malformations are made clear by wood-cuts; but the instruction to apply "antiseptic dressings" after operation in these cases is curious. In prolapsus ani and recti the author reverses their accepted relative frequency in adults and children.

For the chapters on cancer of the rectum and colotomy Dr. Gant has come to the old country, and obtained the help of Mr. Herbert Allingham.

